# Affective and Cognitive Impairments in Rodent Models of Diabetes

**DOI:** 10.2174/1570159X22666240124164804

**Published:** 2024-01-24

**Authors:** Enza Palazzo, Ida Marabese, Serena Boccella, Carmela Belardo, Gorizio Pierretti, Sabatino Maione

**Affiliations:** 1 Department of Experimental Medicine, Pharamacology Division, University of Campania “L. Vanvitelli”, Naples, Italy;; 2 Department of Plastic Surgery, University of Campania “L. Vanvitelli”, Naples, Italy

**Keywords:** Diabetes, animal models, rodents, anxiety, depression, cognitive deficits

## Abstract

Diabetes and related acute and long-term complications have a profound impact on cognitive, emotional, and social behavior, suggesting that the central nervous system (CNS) is a crucial substrate for diabetic complications. When anxiety, depression, and cognitive deficits occur in diabetic patients, the symptoms and complications related to the disease worsen, contributing to lower quality of life while increasing health care costs and mortality. Experimental models of diabetes in rodents are a fundamental and valuable tool for improving our understanding of the mechanisms underlying the close and reciprocal link between diabetes and CNS alterations, including the development of affective and cognitive disorders. Such models must reproduce the different components of this pathological condition in humans and, therefore, must be associated with affective and cognitive behavioral alterations. Beyond tight glycemic control, there are currently no specific therapies for neuropsychiatric comorbidities associated with diabetes; animal models are, therefore, essential for the development of adequate therapies. To our knowledge, there is currently no review article that summarizes changes in affective and cognitive behavior in the most common models of diabetes in rodents. Therefore, in this review, we have reported the main evidence on the alterations of affective and cognitive behavior in the different models of diabetes in rodents, the main mechanisms underlying these comorbidities, and the applicable therapeutic strategy.

## 1 INTRODUCTION

Diabetes mellitus, a metabolic disorder characterized by high glucose levels in the blood, has a high prevalence, continuously increasing in all countries and exceeding 400 million individuals worldwide. Diabetes includes insulin-dependent or type 1 diabetes (T1D), attributed to a lack of insulin, representing 5% of the diabetes cases, and non-insulin-dependent or type 2 diabetes (T2D), characterized by an insensitivity or resistance to insulin, representing 95% of the diabetes cases [1]. Diabetes-associated hyperglycemia causes, over time, micro- and macrovascular complications in organs and tissues, such as the eyes, kidneys, heart, and nerves. The macrovascular complications include myocardial infarction and cerebrovascular diseases, while microvascular complications include nephropathy, retinopathy, and peripheral neuropathy. Macrovascular and microvascular complications of diabetes are described in detail in both clinical and preclinical studies, while complications caused by diabetes in the central nervous system (CNS), although ascertained [2-4], remain less explored. There is a high incidence of psychiatric disorders, such as anxiety, depression, and cognitive impairment, among diabetes patients, especially T2D. Hyperglycemia, impaired insulin signaling, neuroinflammation, and oxidative stress appear to be the underlying mechanisms [5]. The likelihood of developing depression in diabetic patients is approximately double compared to healthy individuals [6]. In particular, among people with T2D, the average prevalence of depression corresponds to 28%, while in the general population, the prevalence of depression is estimated at around 13% [7, 8]. For T1D, the prevalence of depression is 22%, which is lower than for T2D but higher than for the general population [8-10]. Comorbidity of diabetes and depression worsens the prognosis of both diseases and increases the risk for severe cardiovascular complications and mortality [11, 12]. Many studies have investigated the prevalence of anxiety disorders in the diabetic population [13-15]. In an extensive study, the incidence of any anxiety disorder in individuals with diabetes was reported to correspond to 17.7% [14], while in another study, the prevalence reached 47.0% and was also associated with greater disease severity, disability, and poor health state [13]. There is also strong epidemiological evidence linking diabetes to cognitive dysfunctions with highly variable deficits as a function of the type of diabetes and the patient's age [16, 17]. Cognitive decline associated with the diabetic disease increases the risk of developing Alzheimer's disease [17]. The comorbidities of diabetes and affective/cognitive disorders are widely reported in both humans and rodents. Thus, the purpose of this review article is to collect all the available evidence related to the affective/cognitive alterations associated with the main models of diabetes in rodents, the underlying mechanisms, and applicable therapeutic approaches. Such information is essential to elucidate the pathogenesis of human diabetes and its complications, including the neuropsychiatric ones, enabling the screening of antidiabetic molecules for successful translation to patients.

## 2 METHODS

The literature search was performed from the beginning until September 2022, primarily on PubMed, Scopus, and Google Scholar databases. The keywords diabetes, rodents, animal models, anxiety, depression, cognition, affective behavior, cognitive behavior, type 1 diabetes, and type 2 diabetes were used. Relevant articles were selected based on the following criteria: articles investigating affective and cognitive behavior in rodent models of T1D and/or T2D, including studies focusing on the underlying mechanisms or the beneficial effects of molecules proposed as possible therapeutic applications. Studies that defined the possible differences between type 1 and 2 diabetes regarding the risk of affective and/or cognitive complications in rodents and humans were also included. Research and review articles describing diabetic pathology in humans were included in the introduction section. All articles were independently screened by co-authors to determine relevance and were included in the review article if selected and deemed relevant by at least two co-authors. A total of 168 articles were included according to these criteria.

## 3 RODENT MODELS OF DIABETES

Experimental models of type 1 and 2 diabetes in rodents are extensively described, and a detailed description of them is not within the scope of this review [18-20]. Briefly, T1D models include spontaneous (genetic) and chemically induced diabetes. Similar to humans, in these models, T1D develops following the immune-mediated destruction of pancreatic beta cells. Spontaneous models include the non-obese diabetic mice (NOD) and the Akita mice. The Akita mice have an Ins^2+^/C96Y mutation, which is a single nucleotide substitution in the Ins2 gene. Streptozotocin (STZ) is the most widely used agent in chemically induced diabetes. It is a glucose analog that enters pancreatic cells *via* the type 2 glucose transporter (GLUT2), causing beta cell destruction with consequent insulin synthesis and secretion impairment and increased blood glucose levels. Alloxan is another diabetogenic glucose analog causing partial degradation of the beta cells of pancreatic islets. There are several approaches for the development of animal models of T2D, genetic models (both monogenic and polygenic), rich in fat and/or sugar (fructose) diets, administration of low doses of STZ in newborns or combination of STZ with nicotinamide or HFD. While there are models of diabetes in rodents that are not associated with obesity, the majority of models are associated with obesity, reflecting the human condition where obesity predisposes to the development of T2D. Monogenetic models include leptin gene mutations, such as the ob/ob mice (primary leptin deficiency that leads to polyphagia) and the db/db mouse (leptin receptor dysfunction). The Zucker diabetic fat rat (ZDF) also has a missense mutation in the gene that codes for the leptin receptor and spontaneously develops obesity and T2D. WF rats also develop insulin resistance, glucose intolerance, obesity, and T2D. The polygenic models include the Otsuka Long-Evans Tokushima Fatty (OLETF) rat model, showing similar characteristics to human metabolic syndrome with visceral adiposity and the development of insulin resistance. Other polygenic models are the KK mice (mildly obese, hyperleptinaemic, hyperinsulinemic showing insulin resistance), the New Zealand obese (NZO, hyperphagia, hyperinsulinemia, obesity, and leptin resistance), the TallyHo/Jng mice (hyperglycemia, hyperinsulinemia, increased levels of plasma triglycerides, cholesterol and free fatty acid levels) and the NoncNZO10/LtJ mice (liver and skeletal muscle insulin resistance before developing chronic hyperglycemia). The Goto-Kakizaki rat model of T2D is characterized by glucose intolerance and defective insulin secretion but is not associated with obesity. T2D also develops in rodents fed a diet high in fat (HFD) or high in fat and fructose (HFFD) with or without a low dose of STZ administration.

## 4 DEPRESSION-LIKE BEHAVIOR IN RODENT MODELS OF DIABETES

**Fig. (1):**
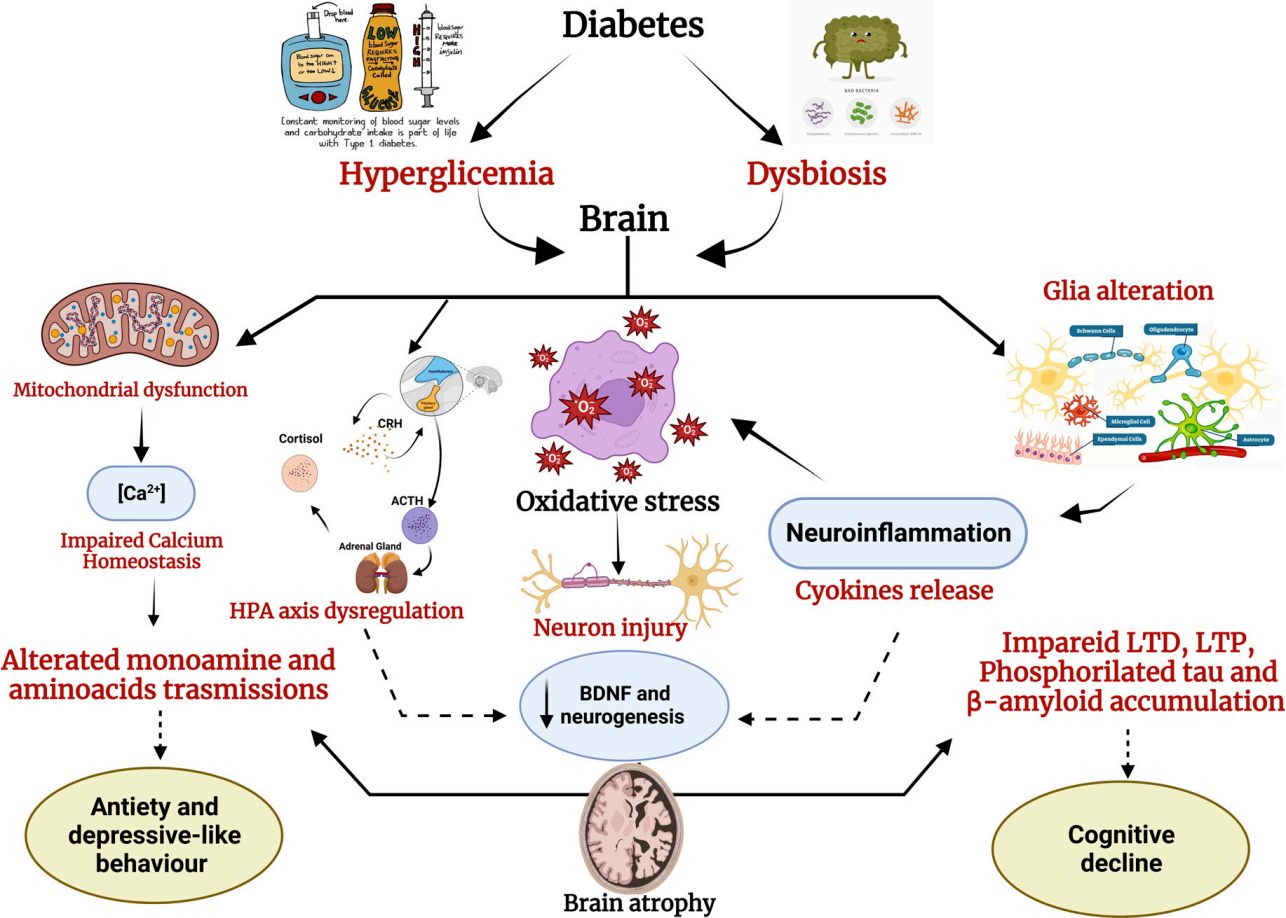
A schematic overview of the main pathophysiological mechanisms implicated in the alterations of cerebral homeostasis contributing to the development of anxiety-like, depression-like behavior, and cognitive disorders in rodent models of diabetes.

Depression is a frequent psychiatric comorbidity found in diabetic patients, who also show an increase in inflammatory biomarkers [21, 22]. Another main mechanism that associates the development of depression with diabetes is oxidative stress from disrupted redox homeostasis [23]. A close correlation between diabetes, oxidative stress, neuroinflammation, and depression was found in animal models of diabetes (Fig. **1**).

### 4.1 T1D

Depression-like behavior associated with increased oxidative stress was observed in the T1D models induced by alloxan or STZ administration. Treatment with imipramine, a tricyclic antidepressant, or hydrogen sulfide, an antioxidant, attenuated both, depression-like behavior and ROS levels [24, 25]. Depression-like behavior in the T1D model induced by STZ has also emerged in other studies [26, 27]. Depression-like behavior improved after treatment with (+/-)-8-hydroxy-2-(di-n-propylamino) tetralin (8-OH-DPAT), a serotonin 1A receptor agonist (5-HT1A) [26] or extract of *Ophiocordyceps formosana*, a *Cordyceps* spp. used in traditional Chinese medicine for anticancer and antidiabetic treatments. *Ophiocordyceps formosana* extract also increased serotonin and dopamine levels in the frontal cortex, striatum, and hippocampus [27]. The levels of serotonin, 5-HT, the major neurotransmitter whose dysregulation is involved in the pathogenesis of depression, were not altered in the prefrontal cortex and amygdala in the STZ-induced T1D model in mice. However, the stress-induced 5-HT release was reduced in the prefrontal cortex but not in the amygdala of T1D mice, thus highlighting that the reduction in stress-induced 5-HT release in T1D mice is site-specific. The same study also reported that diabetic mice showed prolonged freezing in the open platform, corresponding to a sign of fear in the rodents, which was restored by insulin treatment [28].

### 4.2 T2D

A close association between diabetes, depression, and oxidative stress was also found in T2D models. Rats fed a diet rich in fat and fructose (HFFD) showed in addition to obesity and insulin resistance, oxidative stress, depression, anxiety, and cognitive deficits. The beta-caryophyllene, a sesquiterpene, found in many herbs and spices, reduced insulin resistance, oxidative stress, neuroinflammation, and affective/cognitive alterations. However, while the anxiolytic, antioxidant, and anti-inflammatory effects of beta-caryophyllene were mediated by both peroxisome proliferator-activated receptor (PPAR)-alpha and the cannabinoid type 2 receptor (CB2R), the effects on glycemia, depression-like behavior, and memory enhancement appeared to be CB2R-dependent and mediated by an increase of brain-derived neurotrophic factor (BDNF), a neurotrophin that promotes synaptogenesis whose levels and activity are reduced in depressed individuals and are enhanced by antidepressants [29]. The HFD-induced T2D model in rats was associated with dysregulation of glucose and insulin homeostasis and high levels of corticosterone and inflammatory cytokines. In addition, anhedonia, a major symptom of depression, was observed. Treatment with ketamine, a dissociative general anesthetic with potent and rapid antidepressant properties, inhibited anhedonia and restored impaired synaptic activity, glucose, and insulin homeostasis in rats undergoing HFD [30]. Mice undergoing HFD also showed reduced socialization and anhedonia, together with alterations in the gut microbiota and neurotransmitters such as γ-amino-butyric acid (GABA) and neuropeptide Y. These changes did not respond to treatment with imipramine, a tricyclic antidepressant, or sitagliptin, an oral hypoglycemic that inhibits dipeptidyl peptidase IV [31]. Controversial evidence emerged in the genetic model of T2D, the db/db mice, in which both depression-like behavior [32] and the absence of depression-like behavior [33] were reported. However, an increase in the levels of the inflammatory cytokines interleukin-1β (IL-1β), tumor necrosis factor-α (TNF-α), and interleukin-6 (IL-6), and reduced levels of BDNF were found in the hippocampus of db/db mice [33]. In the ZDF rat model, depression-like behavior was closely related to hyperglycemia and stimulation of the auricular vagus nerve was able to revert both alterations [34]. There is also a reciprocal correlation between experimental models of depression and insulin resistance as well as between insulin or oral antidiabetic treatment and antidepressant activity in animal models of T1D and T2D [35-37]. In particular, in a study conducted by Sestile *et al.* [38] in a T2D model induced by the administration of STZ in neonatal rats, insulin showed an antidepressant effect comparable to that of sertraline, an antidepressant of the selective serotonin reuptake inhibitors (SSRIs) class. Therefore, there is a close and reciprocal correlation between depression and animal models of T1D and T2D. Depression-like behavior was associated with oxidative stress in both models, while neuroinflammation appears to be particularly associated with T2D.

### 4.3 Insulin Signaling and Depression-like Behavior

Interestingly, it has been shown that neuronal insulin receptor knock-out (NIRKO) mice have a depression/anxious-like behavior phenotype together with impaired brain dopaminergic neurotransmission [39]. Consistently, the selective ablation of insulin receptors (IR) in the hippocampus and hypothalamus by viral approaches leads to emotional and cognitive disorders [40, 41]. The IR in the brain, apart from being expressed in neurons (IR-A), is also expressed in glial cells (IR-B) [42]. Intriguingly, glial insulin receptor knock-out mice (GIRKO) also showed depression-like behavior [43]. Altogether this evidence highlights that the insulin action in the brain is fundamental for modulating affective behavior; thus, malfunctioning of insulin signaling drives emotional impairments. Cerebral monoamine and BDNF dysregulation are the crucial molecular mechanisms underlying the development of depression. Diabetes is associated with low levels of BDNF and reduced neurogenesis in the hippocampus [44-47], and reduced serotonin levels in the hypothalamus, cortex, and hippocampus [4, 48-54]. The mechanisms underlying low serotonin levels in the brain of diabetic rodents were extensively studied. They appeared to be based on the altered activity of monoamine oxidase enzymes [48, 54], membrane serotonin reuptake transporters [55], 5-HT_1A_ receptors [56, 57], and branched-chain amino acids [58] that compete for the transport of tryptophan, the precursor of serotonin. Consistently, insulin increased the levels of serotonin and its metabolite in the brain [59], while the oral antidiabetic metformin, glyburide, pioglitazone, and sitagliptin increased serotonin levels in the hippocampus and cortex, serotonergic neuronal activity in the dorsal raphe, and mitigated depression-like behavior in rodent models of diabetes [57, 60]. The antidepressant effects of antidiabetic agents in human and animal models of diabetes have been extensively described and reported in the review by Essmat *et al.* [61]. Less clear were the effects of antidepressants on glucose homeostasis: with worsening [62-64], improving [65-69], or no effect [70] being reported in humans. Consistently, animal models of diabetes also led to discordant results, both an improvement in hyperglycemia by the SSRI fluoxetine in a T1D model in the rat [71] and a worsening in hyperglycemia by the SSRIs fluoxetine and fluvoxamine in healthy mice were reported [72].

## 5 ANXIETY-LIKE BEHAVIOR IN RODENT MODELS OF DIABETES

Diabetic patients often show concomitant anxiety disorders, and accumulating evidence indicates that the same occurs in animal models of diabetes.

### 5.1 T1D

Anxiety-like behavior has been widely reported in rats with STZ-induced T1D [73-82]. Some of these studies concurrently examined changes in supraspinal monoamine levels [73, 83]. Anxiety-like behavior was associated with altered levels of monoamines in the prefrontal cortex and hippocampus. In particular, the diabetic rats showed a reduction in the levels of serotonin, noradrenaline, and dopamine in the hippocampus and an increase in noradrenaline, a decrease in serotonin, and no changes in dopamine in the prefrontal cortex. Treatment with cannabidiol, a phytocannabinoid in cannabis plants devoid of psychotropic activity, reverted anxiety-like behavior and monoamine level alterations in the prefrontal cortex and hippocampus [83]. Apart from monoamine dysregulation, a deficiency in the activity of enzymes that regulate the purinergic and cholinergic transmissions was reported in T1D rats [84]. Other studies measured the levels of proinflammatory cytokines and corticosterone associated with metabolic and affective disorders in T1D models in rodents. Cytokine and corticosterone levels increased in the STZ-induced T1D model in the rat [73, 77, 85, 86]. As observed between diabetes and depression, oxidative stress-induced neurotoxicity is the common denominator between diabetes and anxiety. Concomitant anxiety-like behavior and oxidative stress in the STZ-induced model of T1D in the rat have been found in several studies [25, 78, 82, 86-91]. In a recent study by de Lima Silva *et al.* [91], an increase in the memory of fear and difficulty in extinguishing the contextual fear conditioning and anxiety-like behavior were observed after STZ administration. The study also examined oxidative stress showing an increase in lipid peroxidation and a reduction in glutathione levels in the hippocampus and prefrontal cortex of diabetic rats. The same study also showed the efficacy of pregabalin, an antiepileptic that blocks the α2δ subunit of the N-type calcium channel, in reverting anxiety-like behavior and oxidative stress in the hippocampus and prefrontal cortex. Similarly, in the same model of T1D in the rat, anxiety-like behavior proved to be associated with oxidative stress (increased levels of lipid peroxidation and reduced levels of glutathione) in the prefrontal cortex and hippocampus. The treatment with gallic acid, an antioxidant found in tea, grapes, berries, and wine, reverted both anxiety-like behavior and oxidative stress [87]. In addition, other antioxidants, such as garlic (*Allium sativum*), hydrogen sulphide, quercetin (a flavonoid found in many plants and foods, such as red wine, onions, green tea, apples, and berries), resveratrol (a polyphenol found in grapes' skin and seeds), and vitamin E also acted as anxiolytics in the same STZ-induced T1D model in the rat [25, 78, 80, 84, 88, 90] and also inhibited oxidative stress in the brain [25, 88]. Exercise training, whose antioxidant power is well known [80], was also able to mitigate anxiety-like behavior in the same T1D model in the rat [78].

The association between oxidative stress and T1D models was also observed in mice. Mice with T1D exhibited anxiety-like behavior and increased markers of oxidative stress, both of which were reverted by treatment with hydrogen sulfide, which also decreased the cytokine levels [92]. Oxidative stress, high levels of cytokines (TNF-α, IL-1β, IL-6, IL-10), and dysregulation of the proliferation and differentiation of oligodendrocytes and astrocytes were observed in the prefrontal cortex and hippocampus of T1D mice exhibiting concomitantly anxiety-like behavior [93]. Both the metformin, a biguanide oral antihyperglycemic, and fluoxetine mitigated the anxiety-like behavior and the associated neuroinflammation and glial dysregulation [93, 94]. Insulin, glucocorticoids, mineralocorticoids, and type 3 serotonin receptor antagonists were also effective in alleviating anxiety-like behavior associated with STZ-induced T1D in mice [54, 95]. Moreover, the benzodiazepine diazepam synergized with corticosteroid receptor antagonists in alleviating anxiety-like behavior associated with STZ-induced T1D in mice [95]. Aside from some differences between the drug targets addressed for the anxiolytic effect in T1D models, the evidence associating anxious behavior with neuroinflammation, neurotransmitter, neuron and glial alterations [96], and oxidative stress are fairly in line with that found in depressive behavior.

### 5.2 T2D

Anxiety-like behavior was also observed in the T2D model OLETF fatty rat [97, 98] concomitantly with brain atrophy, corticolimbic shrinkage, increased cholecystokinin-positive neurons, and reduced parvalbumin-positive neurons in the infralimbic and prelimbic cortex [98, 99]. Anxiety-like behavior associated with neuroinflammation was observed in the T2D model induced by HFD combined with low doses of STZ in the rat. Parthenolide, an inhibitor of nuclear factor kB (NFκB), ameliorated anxiety-like behavior and neuroinflammation and also rescued GABA and glutamate homeostasis [100]. The HFD in rats induced anxiety-like behavior associated with neuroinflammation, overproduction of IL-1β and IL-6, cerebral oxidative stress, increased lipoperoxidation, inhibition of enzymatic and non-enzymatic antioxidants, and increased ROS levels. Treatment with a chamomile extract reverted the anxiety-like behavior and observed biochemical alterations in the HFD model [101]. In the T2D model induced by a combination of HFD and STZ, it was observed that anxiety-like behavior was associated exclusively with diabetic rats who had concomitant hypertension, but treatment with losartan, an angiotensin II receptor antagonist, although able to reverse hypertension, was not able to modify the anxiety-like behavior [102]. In the T2D model induced by the combination of nicotinamide and low doses of STZ, anxiety-like behavior was mitigated by a combination of diazepam and metformin [103]. In the rat model of T2D induced by HFD and a low dose of STZ, anxiety-like behavior was associated with oxidative stress and low levels of BDNF in the amygdala. The *lactobacillus Plantarum,* a probiotic found in the mouth and gut, and inulin supplements, fermentable non-digestible dietary fibers that can improve metabolic function by intestinal microbiota modulation, or their combination, reduced oxidative stress and anxiety-like behavior and increased BDNF and serotonin levels in the amygdala highlighting the importance of the gut-brain axis, and in particular the gut-amygdala axis, in the psychiatric consequences of T2D [104, 105]. The HFD also produced anxiety-like behavior in mice. The anxiety-like behavior and the associated decreased levels of 5-HT in the hippocampus did not improve following escitalopram treatment but following discontinuation of the HFD [56]. HFD associated with low doses of STZ produced oxidative stress-associated anxiety-like behavior even in mice. These pathological changes improved markedly following exercise [106]. Genetic models of T2D, such as the db/db mice, also showed anxiety-like behavior, increased levels of inflammatory cytokines (IL-1β, TNF-α, and IL-6), and reduced levels of BDNF in the hippocampus [33]. Oxidative stress associated with anxiety-like behavior was reported in the Tsumura Suzuki Obese Diabetes (TSOD) mouse. A diet rich in oleuropein, a phenol found in the leaves of the olive tree, was able to ameliorate oxidative stress and anxiety-like behavior [107]. As depression, neuroinflammation, oxidative stress, and decreased levels of 5-HT and BDNF represent the molecular mechanisms underlying the mutual interaction between diabetes and anxiety. Several agents, including antidepressants, benzodiazepines, antioxidants, antidiabetics, pregabalin, cannabidiol, mineralocorticoid, and glucocorticoid antagonists, or simple exercise have shown anxiolytic effects often restoring the underlying pathological molecular mechanisms.

## 6 COGNITIVE IMPAIRMENTS IN RODENT MODELS OF DIABETES

Diabetes is associated with cognitive deficits and a higher risk of developing Alzheimer's disease. The hyperglycemia-induced neuroinflammation, oxidative stress, neural injury, and apoptosis represent the proposed mechanisms that decrease brain volume and blood-brain barrier efficiency and negatively affect the molecular mechanisms underlying memory and learning in the hippocampus: neurogenesis, long-term potentiation, and long-term depression (LTP and LTD) [3, 108, 109].

### 6.1 T1D

Rodent models of T1D showed evident impairments in cognitive performance [108, 110, 111] that depend on the duration of diabetes and molecular mechanisms, such as those underlying depression-like and anxiety-like behavior: oxidative stress, neuroinflammation, hypothalamic-pituitary-adrenal axis dysregulation, and insulin deficiency [112]. Increased ROS production and poor antioxidant enzyme activity were reported in rats with T1D [113]. Consistently, insulin showed a protective effect against oxidative stress-induced neuron injury [114]. The cognitive impairment in the STZ-induced T1D model also depended strictly on neuroinflammation. Activation of microglia and increased levels of NFkB, TNF-α, IL-1β, IL-2, and IL-6 were found in the cortex and hippocampus of T1D rats [115-117]. In line with the fact that hyperglycemia, neuroinflammation, and oxidative stress are the pathogenetic mechanisms that lead to cognitive deficits in diabetes, the treatment with anti-inflammatories, antioxidants, and C-peptide improved cognitive performance in the T1D models in rats [115, 116, 118]. Cognitive decline in the STZ-induced T1D model is also closely dependent on corticosterone levels and is restored by lowering corticosterone concentration to physiological levels [108, 119]. The elevated levels of glucocorticoids also cause synaptic alterations, such as the depletion of vesicles in moss fibers [120] and the increase and redistribution of neurophysin in the hippocampus [108, 121, 122]. Also, the maladaptive reorganization in the hippocampus of rats with STZ-induced T1D is reverted by lowering the levels of glucocorticoids [108, 110]. Other synaptic alterations in the hippocampus associated with the STZ-induced T1D model consist of astrocyte proliferation and depletion of vesicles and proteins involved in exocytosis at the presynaptic level and an increase in postsynaptic density protein 95 (PSD 95) at the post-synaptic level [120, 121, 123-125]. The absence of insulin in T1D models not only induces cognitive impairment but predisposes and increases the incidence of Alzheimer's disease. Insulin enhances cognitive performance, LTP, LTD, and synaptogenesis [126-130] and increases beta-amyloid and tau-protein clearance [131] in the STZ-induced T1D model in mice. Therefore, the STZ-induced T1D model is also associated with the accumulation of phosphorylated tau and beta-amyloid proteins, pathogenetic markers of Alzheimer's disease [132-135]. Other morphological alterations, such as swelling of neurons and glia in the cortex, hypothalamus, and cerebellum, hippocampal shrinkage, dendrites length shortening, neurogenesis reduction, and apoptosis were also found in the rodent T1D models [96, 136-144]. In addition to morphological alterations associated with cognitive deterioration, alterations in neurotransmitter levels were also observed in T1D models. The levels of serotonin, glutamate, GABA, acetylcholine esterase mRNA, and ATP decreased in the hippocampus and/or cortex [28, 145-148]. In addition, changes in dopamine, calcium homeostasis, Na^+^, and K^+^ currents, N-methyl-D-aspartate (NMDA) receptor expression were also found in the hippocampus of the STZ-induced T1D model [52, 149-151]. The evidence described on depression-, anxiety-like behavior and cognitive deficit in rodent models of T1D is summarized in Table **1**.

### 6.2 T2D

Insulin resistance in the T2D models, as well as insulin deficiency in the T1D models, caused LTP, LTD, synaptogenesis, memory, and learning impairments [108, 152-154]. Oxidative stress also occurred in the Goko-Kakizaki T2D model in rats and drove mitochondrial dysfunction in the brain [114], which was reverted by insulin or antioxidant treatment [113]. Neuroinflammation is another main component underlying neural damage occurring in T2D models in rodents. Activation of macrophages and microglia and increased levels of TNF-α, IL-1β, and IL-6 associated with poor cognitive performance were found in the db/db mice T2D model [33]. The efficacy of rolipram, an inhibitor of phosphodiesterase-4 showing antidepressant activity, in preventing cognitive decline occurred concomitantly with the decrease of the proinflammatory TNF-α and the increase of the anti-inflammatory IL-10 cytokines [155]. Alteration of the normal functioning of the HPA-axis contributes, along with hyperglycemia and insulin resistance, to neural complications associated with cognitive decline in T2D models [130]. Brain insulin receptor malfunctioning in the HFD T2D model caused cytoskeleton and synapse disruption, neural injury, and LTD inefficiency [154, 156]. Insulin resistance was also associated with enhanced tau phosphorylation in the ZDF rat T2D model [135, 157] and impairment of the clearance of β-amyloid and phosphorylated tau proteins owing to reduced activity of the insulin-degrading enzyme (IDE), which also degrades β-amyloid [158]. Reciprocally, the insulin or antioxidant treatment resulted in neuroprotective action and reduced mitochondrial dysfunction [113]. In the HFD T2D model, high levels of leptin were also observed [40]. Although leptin and leptin receptors are abundantly expressed in the hippocampus, where they potentiate LTP in *in vivo* and *in vitro* experiments [159-162], conditions of elevated leptin concentrations, such as those observed in animal models of T2D [40], cause receptor resistance, abnormalities in signal transduction and impairments in synaptic plasticity [163]. The association between disrupted leptin signaling and impaired synaptic plasticity in the hippocampus was demonstrated in the ZDF rat and db/db mouse T2D models [164]. Morphological changes, such as astrogliosis and neurogenesis loss, occurred in the CA1, CA3, and dentate gyrus areas of the hippocampus in the ZDF and Goto Kakizaki rat models of T2D [165, 166]. Neuron apoptosis was also found in T2D models, although less severe than in T1D models [167]. Aberrant calcium homeostasis causing neuron injury and cognitive decline was also found in the HFD T2D model [168]. Moreover, the effects of T2D on neurotransmission, calcium, and ionic currents were less investigated than in T1D models; however, alterations in the molecular mechanisms underlying cognitive impairment in the models of the two types of diabetes appeared to be quite compliant. The evidence described on depression-, anxiety-like behavior and cognitive deficit in rodent models of T2D is summarized in Table **2**.

## CONCLUSION

**Fig. (2):**
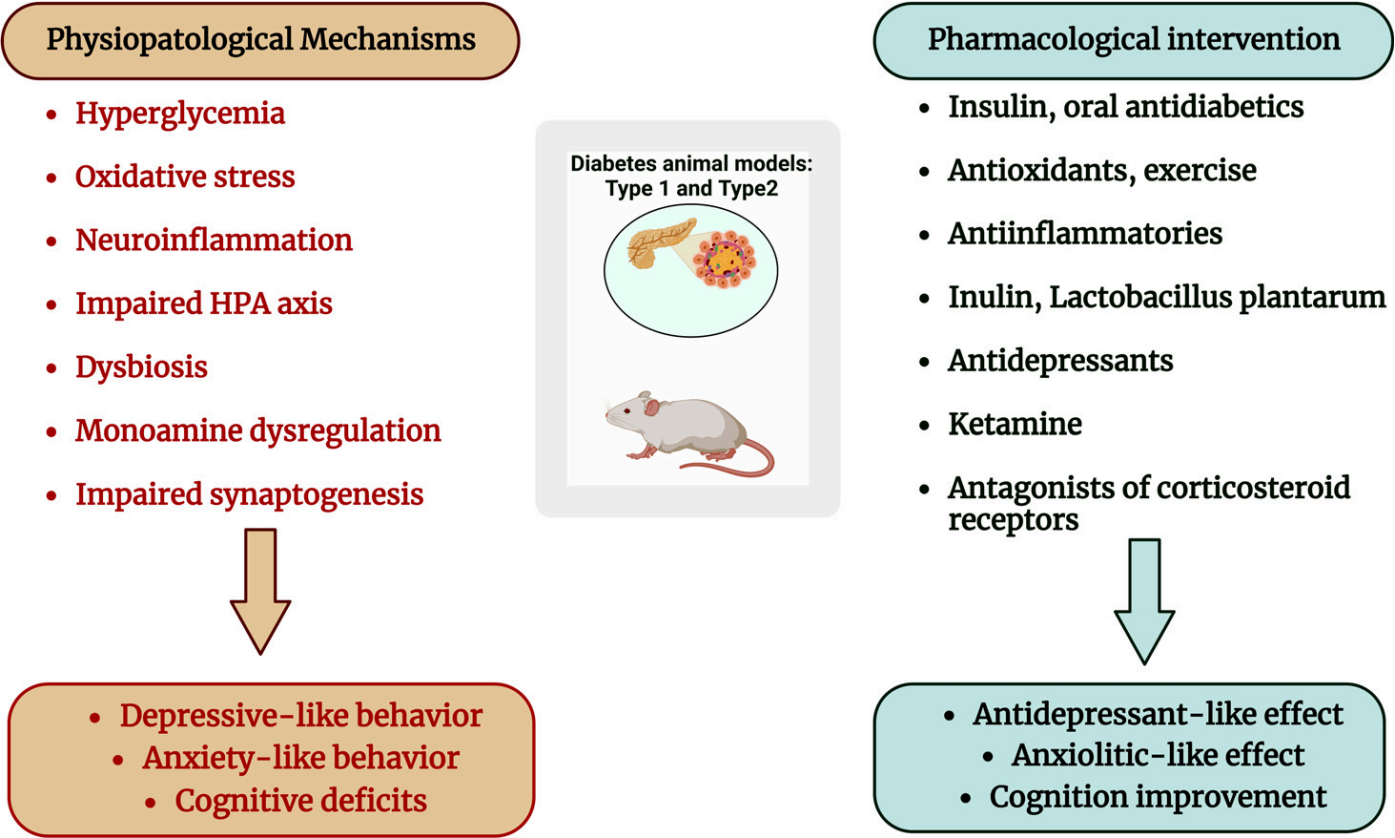
A schematic representation of the different pharmacological strategies directed towards the main pathophysiological mechanisms associated with affective and cognitive disorders in rodent models of type 1 and 2 diabetes.

Animal models that reproduce human pathologies are fundamental for studying the pathophysiological processes underlying the disease and for testing and developing new effective drugs. Such models must therefore reproduce the symptoms and pathological processes associated with the disease. In the case of diabetes, a multi-organ disease associated with macrovascular, microvascular, metabolic, sensory, and affective/cognitive complications, animal models must reproduce the entire sphere of complications found in humans to be valid. Metabolic, micro, and macrovascular sensory alterations are widely treated, while those of affectivity and cognition, although debilitating, are less described. However, it is evident that the two types of diabetes, type 1 and type 2, are associated with affective and cognitive disorders, such as depression, anxiety, and cognitive deficits, not only in humans but also in animal models of diabetes. Therefore, there is a close link between metabolic disorders and CNS alterations. The neurotoxic mechanisms caused by diabetes underlying the neuropsychiatric complications, such as depression, anxiety, and cognitive decline include hyperglycemia, oxidative stress, neuroinflammation, increased glucocorticoid levels, neurotransmitter alterations, and reduced (or absent) insulin signaling. Such molecular mechanisms are quite compliant both in the two types of diabetes and in human and animal models. There is also disturbing evidence that diabetes increases the incidence of Alzheimer's disease, as evidenced by clinical trials and models of diabetes in rodents. The review article highlighted that diabetes has disastrous consequences on the brain functioning. It also highlighted that a multitude of therapeutic approaches has improved the neuropsychiatric component of diabetes, the underlying molecular alterations, and the pathological metabolic dysregulations. These include, in parallel with the neuropathological mechanisms observed in animal models of diabetes, antioxidants, anti-inflammatories, antidepressants, insulin itself, or oral antidiabetics (Fig. **2**), emphasizing proper glycemic control can prevent the neuropsychiatric complications of diabetes. Physical exercise itself, having, among other things, an antioxidant action, improves metabolic syndrome, pathogenetic mechanisms, and related affective disorders. The approach to effective therapies, which aim to improve the entire symptomatological sphere associated with diabetes, starting from animal models, would hopefully lead to more effective and/or better-tolerated therapies.

## Figures and Tables

**Table 1:** Affective and cognitive impairments in rodent models of type 1 diabetes.

**Induction of T1D**	**Species**	**Behavioral Impairments**	**Test**	**Related Evidence**	**References**
Alloxan	Rats	Increased immobility Increased crossing	Forced swim Open field	Treatment with imipramine decreased the immobility and the number of crossings	[24]
STZ	Rats	Increased immobility Reduced open-arm choice	Forced swim, tail suspension Elevated plus maze	Treatment with hydrogen sulfide decreased the immobility, increased open-arm choice, and inhibited oxidative stress in the hippocampus	[25]
STZ	Mice	Increased immobility	Tail suspension	The effects of the 5-HT(1A) receptor agonist, 8-OH-DPAT, and fluoxetine in increasing immobility were attenuated by diabetes	[26]
STZ	Mice	Increased immobility Reduced open-arm choice Reduced central zone retention	Tail suspension Elevated plus maze Open field	*Ophiocordyceps formosana* extract decreased immobility, open-arm choice, central zone retention and increased serotonin and dopamine levels in the frontal cortex, striatum, and hippocampus	[27]
STZ	Mice	Prolonged freezing	Open platform	Insulin treatment decreased freezing	[28]
STZ	Rats	Reduced open-arm choice	Elevated plus maze	Decreased serotonin levels in hippocampus	[73]
STZ	Rats	Reduced open-arm choice Reduced central zone retention	Elevated plus maze Open field	Increased oxidative stress	[74]
STZ	Rats	Increased freezing Increased latency for inhibitory avoidance	Contextual conditioned fear Elevated T maze	Insulin treatment reduced anxiety-like behavior	[75]
STZ	Rats	Increased immobility/freezing behavior	Shock-probe burying	Reversal of the anxiety-like behavior by intra-amygdaloid dopamine D1 receptor blockade	[76]
STZ	Rats	Increased immobility Reduced open-arm choice Reduced central zone retention	Forced swim Elevated plus maze Open field	Reversal of the depression- and anxiety-like behavior by telmisartan	[77]
STZ	Rats	Increased freezing	Open field	Exercise training reduced freezing	[78]
STZ	Rats	Reduced central zone retention Reduced open-arm choice Reduced memory retention Reduced stay in the target quadrant Increased immobility	Open field Elevated plus maze Passive avoidance Morris water maze Forced swim	Ellagic acid treatment attenuated anxiety/ depression-like behaviors, improved exploratory/ locomotor activities, and ameliorated cognitive deficits	[79]
STZ	Rats	Reduction in extinguishing the aversive memory Reduced open-arm choice	Fear conditioning Elevated plus maze	Vitamin E treatment attenuated anxiety-like behavior and fear memory	[80]
STZ	Rats	Reduced central zone retention Reduced open-arm choice Increased latency to feed Increased immobility	Open field Elevated plus maze Novelty suppressed feeding Forced swim	The aqueous extract of the date seed and insulin reverted depression- and anxiety-like behavior	[81]
STZ	Rats	Reduced open-arm choice Increased fear memory and difficulty in extinguishing it	Elevated plus maze Contextual fear conditioning	Pregabalin facilitated the acquisition of the fear extinction memory and produced a later anxiolytic-like effect	[82]
STZ	Rats	Increased immobility Reduced open-arm choice	Forced swim Elevated plus maze	The higher dose of cannabidiol (30 mg/kg) ameliorated depression- and anxiety-like behavior	[83]
STZ	Rats	Impaired memory Reduced latency to step-down from the platform Decreased entries in the open arms	Inhibitory avoidance Open field Elevated plus maze	Quercetin ameliorated memory and anxiety-like behavior	[84]
STZ	Rats	Decreased central zone retention Decreased open-arm choice Increased immobility	Open field Elevated plus maze Forced swim	Loganin treatment showed antidepressant- and anxiolytic-like effect	[85]
STZ	Rats	Decreased central zone retention Decreased open-arm choice Increased immobility Reduced memory retention	Open field Elevated plus maze Forced swim Passive avoidance	Sesamin improved behavioral impairments	[86]
STZ	Rats	Decreased open-arm choice Decreased time spent in the light compartment Increased immobility	Elevated plus maze Light-dark box Modified forced swim	Gallic acid showed an anxiolytic-like effect but not an antidepressant-like effect	[87]
STZ	Rats	Decreased open-arm choice Increased immobility	Elevated plus maze Forced swim	Garlic treatment showed anxiolytic- and antidepressant-like effect	[88]
STZ	Rats	Decreased central zone retention Decreased open-arm choice Decreased exploration of the novel object Reduced memory retention Decreased time spent in the target quadrant	Open field Elevated plus maze Novel object recognition Passive avoidance Morris water maze	*Citrullus colocynthis* improved cognitive and anxiety-like behavior	[89]
STZ	Rats	Increased immobility Decreased open-arm choice	Tail suspension, forced swim Elevated plus maze	Resveratrol treatment ameliorated anxiety-and depression-like behavior	[90]
STZ	Rats	Decreased central zone retention Increased immobility Decreased open-arm choice	Open field Tail suspension Elevated plus maze	Hydrogen sulfide ameliorated the depression- and anxiety-like behavior *via* the phosphoinositide 3-kinase (PI3K)/protein kinase B (AKT) pathway	[91]
STZ	Mice	Reduced alternation Increased immobility Reduced open-arm choice	Radial maze Forced swim Elevated plus maze	Metformin and agmatine ameliorated memory impairment, depression-, and anxiety-like behavior	[93]
STZ	Mice	Reduced alternation Reduced central zone retention Reduced light zone entries Reduced open-arm choice	Y maze Open field Dark/light box Elevated plus maze	Fluoxetine ameliorated anxiety-like behavior and cognitive deficit *via* inhibiting astrocyte activation and repairing the oligodendrocyte damage	[94]
STZ	Mice	Increased burying	Burying behavior	MR or GR antagonists synergized with diazepam to induce anxiolytic-like effects	[95]
STZ	Rats	Decreased novel object exploration Decreased time spent in the target quadrant	Novel object recognition Morris water maze	Normalization of corticosterone levels ameliorated cognitive behavior	[108]
STZ	Rats	Impaired performance	Morris water maze	Insulin treatment prevented (but not reversed) learning impairment	[110]
STZ	Rats	Decreased performance	Can test	The test showed impairments of both, reference and working memory	[111]
STZ	Rats	Increased transfer latency Decreased ability to reach the platform	Morris water maze	The insulin-tocotrienol combination treatment ameliorated cognitive deficit	[115]
BB/Wor	Rats	Prolonged latency	Morris water maze	The c-peptide replacement restored cognitive deficit	[116]
STZ	Rats	Poor learning	14-unit T-maze	Prevention of corticosterone increase prevented impairment in complex maze learning	[119]

**Table 2:** Affective and cognitive impairments in rodent models of type 2 diabetes.

**T2D Model**	**Species**	**Behavioral Impairments**	**Test**	**Related Evidence**	**References**
High fat/fructose diet	Rats	Increased immobility Decreased central zone and light area retention Decreased open-arm choice Decreased novel arm entries	Forced swim Open field Light/dark box Elevated plus maze Y maze	Beta-caryophyllene alleviated oxidative-stress, neuroinflammation and behavioral impairments. The anxiolytic, anti-oxidant and anti-inflammatory effects were mediated by both PPAR-γ and CB2R.	[29]
High-fat diet	Rats	Increased latency to feed Decreased sucrose consumption Decreased female sniffing urine Reduced entries in the center zone Decreased open-arm choice Decreased novel object exploration	Novelty suppressed feeding Sucrose preference Female urine sniffing Open field Elevated plus maze Novel object recognition	Ketamine reversed behavioral deficits. The inhibitor of the purinergic P2X7 receptor ameliorated anxiety	[30]
High-fat diet	Mice	Decreased socialization Decreased sucrose consumption	Three-chamber social interaction Sucrose preference	The DPP-4 inhibitor sitagliptin and the TCA antidepressant imipramine did not ameliorate the depression-like social interaction and anhedonia	[31]
db/db	Mice	Increased immobility Increased open-arm choice Disrupted pre-pulse inhibition	Forced swim Elevated plus maze Pre-pulse inhibition	Depression-like behavior was found in both, juvenile and adult groups while the psychosis-like symptoms showed an age-dependent progression	[32]
db/db	Mice	Reduced entries in the center zone Reduced open-arm choice Decreased novel arm entries Decreased novel object exploration	Open field Elevated plus maze Y-maze Novel object recognition	The expression of the inflammatory cytokines, interleukin-1β, tumor necrosis factor-α and interleukin-6 were increased while the expression of brain-derived neurotrophic factor (BDNF) were reduced in the hippocampus but not the hypothalamus	[33]
ZDF	Rats	Increased immobility	Forced swim	Ameliorative effect by transcutaneous auricular vagus nerve stimulation	[34]
STZ in newborns	Rats	Increased immobility	Forced swim	Insulin and sertraline showed antidepressant-like effect	[38]
OLETF	Rats	Reduced open-arm choice Reduced time spent in the light box	Elevated plus maze Dark/light box	The missing CCKA receptor in OLETF rats produced anxiogenic-like behavior	[97]
OLETF	Rats	Reduced time spent in the central zone	Open field	Corticolimbic area hypotrophy	[98, 99]
High-fat diet/STZ	Rats	Increased latency and path to reach the platform Decreased avoidance Reduced open-arm choice Reduced time in the central zone	Morris water maze Passive avoidance Elevated plus maze Open field	Parthenolide ameliorated anxiety-like behavior and cognitive deficit and restored GABA and glutamate homoeostasis.	[100]
High-fat diet	Rats	Decreased open-arm choice Reduced time spent in the central zone Reduced time spent in the light box	Elevated plus maze Open field Light/dark box	Treatment with chamomile extract reverted anxiety-like behavior	[101]
High-fat diet/STZ	Rats	Decreased open-arm choice Decreased ambulation, distance and rearing Decreased recognition memory	Elevated plus maze Open field Novel object recognition	Losartan ameliorated cognitive deficit but did not affect anxiety-like behavior	[102]
STZ/nicotinamide	Rats	Decreased open-arm choice	Elevated plus maze	A combination of metformin and diazepam ameliorated anxiety-like behavior	[103]
High-fat diet/STZ	Rats	Decreased open-arm choice Increased immobility	Elevated plus maze Forced swim	*Lactobacillus plantarum*, inulin, or their combination ameliorated anxiety- and depression-like behavior	[104]
High-fat diet/STZ	Mice	Decreased central zone entries Decreased light box entries Decreased open-arm choice	Open field Light-dark box Elevated plus-maze	Swimming exercise reversed anxiety-like behavior	[106]
TSOD	Mice	Decreased central zone retention and increased freezing	Open field	Oleuropein-containing supplement ameliorated anxiety like-behavior	[107]
db/db	Rats	Impaired novel object exploration	Novel object recognition	Normalization of corticosterone levels reversed the cognitive deficit	[108]
High-fat diet	Rats	Impaired alternation	Variable-interval delayed alternation	Cognitive decline was associated with saturated fatty acid intake	[152]
ZDF	Rats	Impairment alternation at longer intervals	Variable-interval delayed alternation	The glucose transporter, GLUT4, expression was reduced in the hippocampus	[153]
High-fat diet/STZ	Rats	Increased platform training latency Decreased time spent in the target quadrant	Morris water maze	Treatment with rolipram improved cognitive performance	[155]

## LIST OF ABBREVIATIONS

BDNF: Brain-derived Neurotrophic Factor
CB2R: Cannabinoid Type 2 Receptor
CNS: Central Nervous System
SSRIs: Serotonin Reuptake Inhibitors
STZ: Streptozotocin
T1D: Type 1 Diabetes
TSOD: Tsumura Suzuki Obese Diabetes
ZDF: Zucker Diabetic Fat Rat
